# A Case of Dropsy

**Published:** 1880-12

**Authors:** J. F. Goldman

**Affiliations:** Huntsville, Ala.


### Selections.

### A CASE OF DROPSY.

By J. F. GOLDMAN, M.D., HUNTSVILLE, ALA.

August 6th was called to see Miss J. B , who had been for many months the victim of this dread disease. Her whole system was involved; but, more especially, her lower extremities and bowels. The infiltration was so great that her abdomen had become painfully immense. She had exhausted the list of domestic and patent remedies, and had been under the hands of several good physicians; the last one (as he informed me) counseled tapping, as her only relief.

On being called, and learning these facts, I was convinced that the usual remedies had been exhausted, and something out of the regular routine must be done.

Having a spirit-lamp of a more than ordinary size burner, I determined to sweat the surplus water out of my patient. After removing all clothing, she was seated in a common split-bottom chair and wrapped in two blankets —the blankets passing around and including the chair. The alcohol lamp was lighted and set under the chair. In a few minutes my patient had "rivers of waters" pouring from her. The bath was continued about twenty minutes, when my patient was put to bed with the blankets still around her, and thus continued the sweating in bed. I gave her a drachm doses of fl. ext. jaborandi, and further reduced the supply of water in her system by opening the salivary glands and stimulating the kidneys to extra activity.

Result:—The second Sunday after beginning treatment my patient put on her corset (a thing she had never hoped to do again) and went to Sunday-school, to the amazement of all her friends.

I gave her two baths per day, allowed her to drink ice water during the time of each bath, and gave her hot drinks on going to bed.

Before treatment her skin had the appearance of old parchment, and her face was covered with dark blotches. All this was changed in a few days to the softness and whiteness of infancy.—*Medical Brief.*

